# Personality functioning and self-disorders in individuals at ultra-high risk for psychosis, with first-episode psychosis and with borderline personality disorder

**DOI:** 10.1192/bjo.2023.530

**Published:** 2023-08-11

**Authors:** Maria Gruber, Johanna Alexopoulos, Stephan Doering, Karin Feichtinger, Fabian Friedrich, Miriam Klauser, Barbara Hinterbuchinger, Zsuzsa Litvan, Nilufar Mossaheb, Karoline Parth, Antonia Wininger, Victor Blüml

**Affiliations:** Department of Psychoanalysis and Psychotherapy, Medical University of Vienna, Vienna, Austria; and Department of Psychiatry and Psychotherapy, Clinical Division of Social Psychiatry, Medical University of Vienna, Vienna, Austria; Department of Psychoanalysis and Psychotherapy, Medical University of Vienna, Vienna, Austria; Department of Psychiatry and Psychotherapy, Clinical Division of Social Psychiatry, Medical University of Vienna, Vienna, Austria

**Keywords:** Personality functioning, Structured Interview for Personality Organization (STIPO), psychotic personality organisation, self-disorders, at-risk mental state for psychosis

## Abstract

**Background:**

Assessment of personality functioning in different stages of psychotic disorders could provide valuable information on psychopathology, course of illness and treatment planning, but empirical data are sparse.

**Aims:**

To investigate personality functioning and sense of self in individuals at ultra-high risk (UHR) for psychosis and with first-episode psychosis (FEP) in comparison with a clinical control group of individuals with borderline personality disorder (BPD) and healthy controls.

**Method:**

In a cross-sectional design, we investigated personality functioning (Structured Interview of Personality Organization, STIPO; Level of Personality Functioning Scale, LPFS) and disturbances of the basic self (Examination of Anomalous Self-Experience, EASE) in 107 participants, comprising 24 individuals at UHR, 29 individuals with FEP, 27 individuals with BPD and 27 healthy controls.

**Results:**

The UHR, FEP and BPD groups had moderate to severe deficits in personality organisation (STIPO) compared with the healthy control group. Self-functioning with its subdomain (facet) ‘self-direction’ (LPFS) was significantly worse in participants with manifest psychosis (FEP) compared with those at-risk for psychosis (UHR). The FEP group showed significantly worse overall personality functioning than the UHR group and significantly higher levels of self-disturbance (EASE) than the BPD group, with the UHR group lying between these diagnostic groups. Hierarchical cluster analysis based on the seven STIPO domains yielded three clusters differing in level of personality functioning and self-disturbances.

**Conclusions:**

Our data demonstrate that psychotic disorders are associated with impaired personality functioning and self-disturbances. Assessment of personality functioning can inform treatment planning for patients at different stages of psychotic disorder.

The interaction between psychosis and personality has been an area of conceptual investigation since the times of Kraepelin, Bleuler and Freud. Despite the potential relevance for aetiology, course of illness, treatment planning and outcome, especially in at-risk states and early psychosis, there is a scarcity of empirical research into this complex relationship.^1–4^ Impaired personality functioning and pathological personality traits, as for example captured in the diagnosis of a cluster A personality disorder (paranoid, schizoid and schizotypal), may be present before the onset of psychotic illness and could be considered as a premorbid predisposition or antecedents for psychotic disorders.^2,4^ Disturbances of the pre-reflective basic self have been considered to constitute the core characteristic of schizophrenia in the continental European phenomenological tradition.^2,5,6^ Furthermore, deterioration of personality functioning has been linked to the progress of the psychotic process, affecting the long-term outcome of schizophrenia.^7,8^ Meanwhile, psychosis-like experiences have also been found in non-clinical samples,^3^ suggesting that psychotic symptoms may occur at all levels of personality functioning and in different psychiatric disorders.^9^

These nosological and conceptual issues are also reflected in the long-standing debate about the status of borderline personality disorder (BPD) in relation to psychotic disorders,^10^ as psychotic symptoms are known to be frequent in BPD^11^ and, in the form of ‘transient, stress-related paranoid ideation’ (DSM-5) or ‘psychotic-like features’ (ICD-11), form part of the diagnostic criteria of BPD.^12^ To clarify the diagnostic boundaries between these disorders more detailed psychopathological investigations focusing on core pathognomonic characteristics have been called for.^10^

## Personality functioning

The publication of the new editions of the diagnostic classification systems DSM-5^13^ and ICD-11^14^ has sparked an increase in interest in personality functioning in the area of personality disorder research and beyond.^15^ Aspects of self (e.g. identity, self-worth, accuracy of self-view, self-direction) and interpersonal functioning (e.g. ability to develop and maintain close and mutually satisfying relationships, ability to understand others’ perspectives and to manage conflict in relationships) are now considered as core components of personality disorders in the alternative model of personality disorders (AMPD) of DSM-5^13^ and ICD-11.^14^

These models of personality functioning in DSM-5 and ICD-11 converge with long-standing psychodynamic conceptualisations.^16,17^ One influential psychodynamic model to conceptualise personality functioning^16^ comprises three basic levels: neurotic, borderline and psychotic personality organisation. These levels of personality organisation are distinguished by differences in identity integration, maturity of defence mechanisms, the capacity for reality testing, and the integration of aggression and moral values. A neurotic level of personality organisation is defined by an integrated identity, relatively mature defence mechanisms, (e.g. anticipation) and intact reality testing. Borderline personality organisation is characterised by identity diffusion and the use of primitive defence mechanisms (mainly splitting and projective identification) with intact capacity for reality testing. Psychotic personality organisation shows worse functioning in all domains (severe identity diffusion, extensive use of primitive defence mechanisms) and impaired reality testing (corresponding to a loss of differentiation between self and object representations).^18^

## Self-functioning and self-disorders

Another framework for the study of disturbances of self-functioning (‘self-disturbances’) is provided by modern phenomenological psychiatry, which distinguishes between different levels of selfhood.^19^ The ‘basic self’ designates the fundamental level of selfhood that is implied in the awareness that under normal conditions all experiences are experienced as ‘my experience’ (first-person quality of experience).^20^ The basic self is the prerequisite for the higher-order ‘narrative self’, which designates language-involving and autobiographical aspects of the self that are related to areas such as habits, style and preferences.^21^ The presence of basic self-disturbances has been shown to differentiate between schizophrenia spectrum disorders and other psychotic disorders^22^ and they were found to be temporally enduring, preceding and predicting the onset of psychosis^23^ and persisting after remission of a psychotic episode.^19,20^ Consequently, distortions of the basic self are again included as defining features of schizophrenia in ICD-11.^24^

There are interesting points of convergence between the psychodynamic approach to personality assessment and the phenomenological approach. The in-depth psychopathological evaluation of the level of selfhood affected has been proposed as crucial for the differential diagnosis between borderline personality disorder and schizophrenia spectrum disorders.^25^ In this sense, identity diffusion, a pathognomonic characteristic of borderline personality organisation, has been suggested to correspond to disturbances in higher levels of selfhood, i.e. the ‘narrative’ self, but there are only preliminary empirical data to support this hypothesis.^21^ Meanwhile, the concept of psychotic personality organisation with a severe disorganisation of identity should converge with disorders of the ‘basic’ self in schizophrenia spectrum disorders.

## Aims

The investigation of personality functioning and self-disorders is of particular interest in individuals at ultra-high risk for psychosis (UHR) and in the early stages of psychosis. A careful assessment of these aspects might provide valuable information on the transition from at-risk mental states to manifest psychosis and help in identifying potential targets for specific treatment modalities.

Considering all the above, it was the aim of this research project to investigate personality functioning and self-disorders in individuals at UHR for psychosis and with first-episode psychosis (FEP) in comparison with a clinical control group of individuals with borderline personality disorder (BPD) and a group of healthy controls.

Given the scarcity of empirical studies on the topic, this study was of a primarily exploratory nature. Deriving from mainly conceptual considerations^18^ and some preliminary empirical evidence^26^ we hypothesised to find the most severe impairment in personality functioning and basic self-disturbances in patients with manifest psychotic disorders (FEP).

## Method

### Participants

Participants were recruited from the Department of Psychiatry and Psychotherapy and the Department of Psychoanalysis and Psychotherapy of the Medical University of Vienna and from several psychiatric departments of hospitals situated in Vienna and surroundings. The control group was recruited via announcements.

All participants needed to meet the following inclusion criteria: age ≥18 years, sufficient command of German and cognitive capability for an adequate understanding of the interviews. Exclusion criteria were psychiatric symptoms due to any organic condition or acute intoxication. Additional exclusion criteria for each study group are listed below.

Out of 170 individuals who agreed to participate in the study after initial information, 118 individuals completed all interview appointments. Some individuals with very high psychotic symptom scores (Positive and Negative Syndrome Scale (PANSS) >95 points) were asked to postpone the interviews to wait for symptom remission after antipsychotic treatment (see ‘Individuals with first-episode psychosis’ below).

After data entry and verification, 11 participants were excluded because of missing data on the main instruments (Structured Clinical Interview for DSM-IV; Structured Interview of Personality Organization; Level of Personality Functioning Scale; Examination of Anomalous Self-Experience) or for not fulfilling the inclusion/exclusion criteria (two participants did not fulfil inclusion criteria owing to psychotic symptoms lasting longer than 5 years, one participant was excluded owing to a PANSS score >95 points, and one was excluded owing to a too high Global Severity Index). Valid data of 107 participants (24 UHR, 29 FEP, 27 BPD and 27 healthy controls) could finally be included for statistical analyses.

The authors assert that all procedures contributing to this work comply with the ethical standards of the relevant national and institutional committees on human experimentation and with the Helsinki Declaration of 1975, as revised in 2008. All procedures involving human subjects/patients were approved by the Ethics Committee of the Medical University of Vienna (approval number: 1116/2015). Written informed consent was obtained from all participants.

#### Individuals at ultra-high risk (UHR) for psychosis

The UHR participants were recruited from a specialised early psychosis unit where standard assessments include the 16-item Prodromal Questionnaire (PQ-16), the Comprehensive Assessment of At-Risk Mental States (CAARMS),^27^ laboratory tests, electroencephalogram (EEG) and cranial magnetic resonance imaging (MRI). The CAARMS interview allows for the identification of three groups at UHR for psychosis: (a) an attenuated psychotic symptoms group; (b) a brief limited intermittent psychotic symptoms group; and (c) a genetic risk and deteriorating state group. In all three groups a significant decrease in psychosocial functioning over the past year for a period of at least 1 month is needed. In addition, the Schizophrenia Proneness Instrument, Adult version (SPI-A)^28^ was administered to those individuals who were suspected of having basic symptoms on clinical examination for the operationalised assessment of the cognitive-perceptual (COPER) at-risk criterion and the cognitive disturbances (COGDIS) high-risk criterion. Following recommendations of the European Psychiatric Association, clinical high risk was identified when fulfilling either UHR or COGDIS criteria or both.^29^

#### Individuals with first-episode psychosis (FEP)

The FEP group comprised patients at first admission for a psychotic episode (with a maximum duration of the presence of psychotic symptoms of 5 years^30^) who were screened and diagnosed with schizophrenia spectrum, schizoaffective or affective psychotic disorders using the Structured Clinical Interview for DSM-IV Axis I Disorders (SCID-I).^31^ Owing to the known diagnostic instability of first-episode psychosis^32^ and the increasing recognition of the dimensional nature of psychotic disorders^33^ we included both affective and non-affective disorders in our FEP sample.^34^ Additionally, the Positive and Negative Syndrome Scale (PANSS)^35^ was used to assess the acute severity of symptoms that might affect the measurement of personality functioning. Individuals with a score of more than 95 were classified as ‘currently severely ill’ and excluded.

#### Individuals with borderline personality disorder (BPD)

The BPD control group borderline personality disorder was diagnosed via the SCID-II (Axis II disorders).^31^ The presence of a psychotic disorder was excluded by the application of SCID-I and patients were screened for the risk of psychosis using the PQ-16.^36^

#### Healthy control group

The SCID-I and SCID-II^31^ were used to exclude any diagnosis of a current psychiatric disorder and participants were excluded with a Global Severity Index >0.32 on the Brief Symptom Inventory.^37^

### Measures

All interviews were conducted by psychiatrists or clinical psychologists who were trained in administration of each interview and had passed inter-rater reliability testing (intraclass correlation coefficient for the overall Structured Interview of Personality Organization rating: 0.760).

#### Structured Interview of Personality Organization (STIPO)

The STIPO^38^ is grounded in Kernberg's model of personality organisation.^16,17^ The German version^39^ consists of 100 items, seven domains and specific subdomains: 1 Identity, subdivided into 1A Capacity to invest, 1B Sense of self (1B is further subdivided into (a) Coherence and continuity, (b) Self valuation) and 1C Sense of others; 2 Object relations, subdivided into 2A Interpersonal relationships, 2B Intimate relationships and sexuality and 2C Internal working model of relationships; 3 Primitive defences; 4 Coping/rigidity; 5 Aggression, subdivided into 5A Self-directed aggression and 5B Other-directed aggression; 6 Moral values; and 7 Reality testing and perceptual distortions. Each domain is rated on a five-point scale from healthy functioning (1 point) to severe impairment (5 points). Finally, the overall level of personality organisation is assessed, ranging from a normal level to severely impaired personality functioning: level 1, normal; 2, ‘neurotic 1’; 3, ‘neurotic 2’; 4, ‘borderline 1’; 5, ‘borderline 2’; and 6, ‘borderline 3’. Satisfactory reliability and validity have been demonstrated.^39,40^

#### Level of Personality Functioning Scale (LPFS)

The LPFS^41^ is part of DSM-5, Section III (Emerging Measures and Models)^13^ and it assesses personality functioning on a five-point scale from healthy functioning (scored 0) to extreme impairment (scored 4) in two domains, which are subdivided into four subdomains (facets): ‘Self-functioning’, with the subdomains ‘Identity’ and ‘Self-direction’; and ‘Interpersonal functioning’, with the subdomains ‘Empathy’ and ‘Intimacy’. The German version of the LPFS has good psychometric properties^42^ and was rated on the basis of the STIPO interviews.^43^

#### Examination of Anomalous Self-Experience (EASE)

The EASE^44^ was designed for the examination of anomalous subjective self-experiences, which are characteristic of schizophrenia spectrum disorders and prodromal states of psychotic disorders.^20^ The EASE consists of 57 items in five domains: cognition and stream of consciousness; self-awareness and presence; bodily experiences; demarcation/transitivism; and existential reorientation. Each item is rated dichotomously (present/absent). Good to excellent psychometric properties have been shown.^45^

#### Structured Clinical Interview for DSM-IV (SCID)

The SCID^31^ was used to diagnose psychiatric disorders according to DSM-IV Axis I (SCID-I) and personality disorders according to DSM-IV Axis II (SCID-II).

#### 16-item Prodromal Questionnaire (PQ-16)

This self-report questionnaire is a brief version of the PQ^46^ for routine screening for psychosis risk in general help-seeking populations. Cronbach's alpha for the total PQ-16 score was 0.774.^36^

### Statistics

Kolmogorov–Smirnov and Levene tests were employed to ensure the normality and homogeneity of variances. Non-parametric tests (Kruskal–Wallis tests, Mann-Whitney *U*-tests) were used for the comparison of the non-normally distributed overall levels and for all seven domains of the STIPO in the unrelated samples (UHR, FEP, BPD and healthy controls). Results on the overall EASE score were normally distributed and analysed using a one-way analysis of variance (ANOVA). *Post hoc* analysis was carried out using independent-sample *t*-tests. Adjustments for multiple testing were done using the Benjamini–Hochberg procedure with a false discovery rate of 15%. Relationships between STIPO and LPFS or EASE total scores were assessed with Spearman's rho correlation coefficients (*ρ*).

An agglomerative hierarchical clustering technique was applied to group the patients (UHR, FEP and BPD) according to the seven STIPO domains, irrespective of psychiatric diagnosis (the healthy control group was not included in the cluster analysis). Ward's minimum variance method, which has the highest accuracy of the clustering methods,^47^ was used to combine pairs of clusters at each step. It starts with each individual participant being one cluster and continues until all clusters are combined into a single cluster. Each new step is reached by minimising variance using the sum of square index, respectively. Each of the 79 patients (one patient's data had to be excluded due to partially incomplete data) were placed in their own cluster and then progressively clustered with others according to the STIPO domain. To assess how the three patient groups would be distributed based on the STIPO domains, we opted *a priori* for a three-cluster solution.

Analyses were carried out using SPSS 28.0 for MacOS.

## Results

### Sample characteristics

Basic demographic data are shown in Table 1.

**Table 1:** Demographic data

	Participants			
	UHR	FEP	BPD	Healthy controls
*n*	24	29	27	27
Female gender, *n* (%)	12 (50)	15 (51.7)	25 (92.6)	22 (81.5)
Age, years: mean (s.d.)	22.55 (2.97)	24.15 (3.70)	28.40 (6.49)	30.71 (11.68)
Treatment setting, *n* (%)				
In-patient	14 (58.3)	26 (89.7)	18 (66.7)	
Day patient		1 (3.4)		
Out-patient	10 (41.7)	2 (6.9)	9 (33.3)	
Non-treatment setting				27 (100)
Partnership, *n* (%)	7 (29.2)	8 (27.5)	12 (44.4)	22 (81.5)
Highest educational level, *n* (%)				
Pre-secondary school	6 (25)	12 (41.3)	5 (18.4)	2 (7.4)
Secondary school	12 (50)	11 (37.9)	11 (40.7)	15 (55.6)
University	4 (16.7)	4 (13.8)	9 (33.3)	10 (37)
Employed or in training, *n* (%)	19 (66.7)	20 (68.9)	15 (48.1)	26 (96.3)
Psychopharmacotherapy, *n* (%)	20 (83.3)	29 (100)	21 (77.8)	0
Psychiatric treatment, *n* (%)	19 (79.2)	28 (96.6)	20 (74.1)	0
Psychotherapeutic treatment, *n* (%)	15 (62.5)	18 (62.1)	14 (51.9)	1 (3,7)

BPD, borderline personality disorder; FEP, first-episode psychosis; UHR, ultra-high risk for psychosis.

The distribution of male and female participants was almost equal in the UHR and FEP groups, but the gender distribution was not balanced in the BPD and healthy control groups. An equal distribution of levels of personality functioning and abnormal self-experiences was observed in male and female participants with FEP and UHR (*P* > 0.05).

Diagnoses according to DSM-IV^48^ are given in Table 2.

**Table 2:** Axis I and Axis II diagnoses according to DSM-IV

	Participants, *n* (%)			
	UHR (*n* = 24)	FEP (*n* = 29)	BPD (*n* = 27)	Healthy controls (*n* = 27)
**Axis I**				
Any Axis I disorder	24 (100)	29 (100)	24 (78.5)	0
Bipolar I disorder*–*	0	7 (24.1)	1 (3.7)	0
Bipolar II disorder	3 (2.97)			0
Depressive disorder	14 (58.3)	13 (44.8)	16 (59.3)	0
Anxiety disorder	15 (62.5)	6 (20.7)	8 (29.6)	0
Phobia	9 (37.5)	7 (24.1)	6 (22.2)	0
Substance-related disorder	9 (37.5)	15 (51.7)	10 (37.0)	0
Eating disorder	3 (12.75)	2 (6.9)	7 (25.9)	0
Schizophrenia spectrum disorder	2 (8.3)	21 (72.4)	0	0
Psychotic disorder unspecified	0	1 (3.2)	0	0
Obsessive-compulsive disorder	3 (12.75)	4 (13.8)	1 (3.7)	0
Somatoform disorder	3 (12.75)	0	3 (11.1)	0
Developmental disorder	1 (4.2)	1 (3.4)	0	0
**Axis II Personality disorders**				
Any Axis II disorder	17 (70.8)	19 (65.5)	27 (100)	0
Cluster A				
Paranoid	3 (12.5)	6 (20.7)	4 (14.8)	0
Schizoid	1 (4.2)	4 (13.8)	0	0
Schizotypal	5 (20.8)	8 (27.6)	0	0
Cluster B				
Antisocial	2 (8.3)	2 (6.9)	0	0
Borderline	5 (20.8)	1 (3.4)	27 (100)	0
Histrionic	1 (4.2)	1 (3.4)	1 (3.7)	0
Narcissistic	1 (4.2)	1 (3.4)	2 (7.4)	0
Cluster C				
Avoidant	7 (29.2)	6 (20.7)	6 (22.2)	0
Dependent	4 (16.7)	4 (13.8)	2 (7.4)	0
Obsessive–compulsive	4 (16.7)	1 (3.4)	4 (14.8)	0
**Other**				
Depressive	2 (8.4)	2 (6.9)	7 (25.9)	0
Negativistic	8 (33.4)	4 (13.8)	1 (3.7)	0

BPD, borderline personality disorder; FEP, first-episode psychosis; HC, healthy controls; UHR, ultra-high risk for psychosis.

### Level of personality functioning

#### Structured Interview of Personality Organization (STIPO)

The groups differed significantly with respect to their overall level of personality organisation (χ²(3) = 65.496, *P* < 0.001; Table 3). Patients in the UHR (mean 4.29, s.d. = 0.908), BPD (mean 4.70, s.d. = 0.542) and FEP groups (mean 4.83, s.d. = 1.002) showed intermediate to severe impairment in overall personality functioning compared with healthy controls (mean 1.63, s.d. = 0.565; for all comparisons *P* < 0.001). Pairwise comparison further revealed a significant difference in the level of personality functioning between the UHR and FEP groups (*U* = 236.50, *P* = 0.037), with those in the BPD group lying in between these groups but without significant differences from the UHR (*U* = 233.50, *P* = 0.062) and FEP groups (*U* = 346.50, *P* = 0.427).

**Table 3:** Structured Interview of Personality Organization (STIPO) level of personality functioning in the four different groups (*n* = 107)

STIPO level	UHR (*n* = 24), *n* (%)	FEP (*n* = 29), *n* (%)	BPD (*n* = 27), *n* (%)	Healthy controls (*n* = 27), *n* (%)
1 normal				11 (40.7)
2 neurotic 1				15 (55.6)
3 neurotic 2	5 (20.8)	3 (10.3)		1 (3.7)
4 borderline 1	9 (37.5)	8 (27.6)	9 (33.3)	
5 borderline 2	8 (33.3)	9 (31.0)	17 (63.0)	
6 borderline 3	2 (8.3)	9 (31.0)	1 (3.7)	

BPD, borderline personality disorder; FEP, first-episode psychosis; UHR, ultra-high risk for psychosis.

**Fig. 1:**
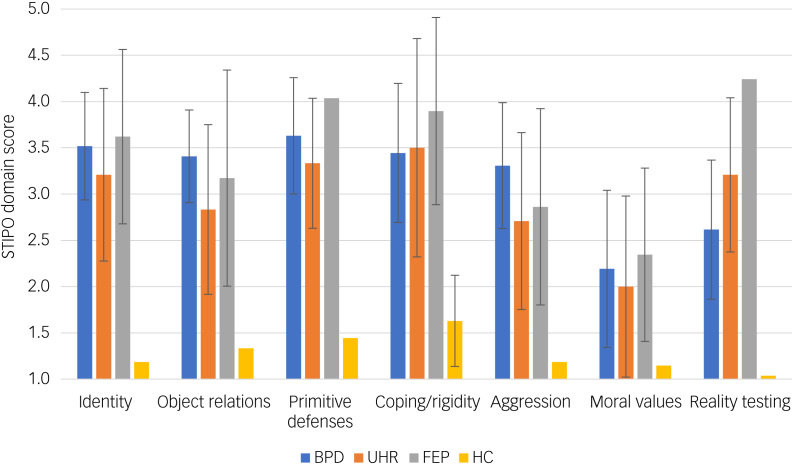
Bar chart of the Structured Interview of Personality Organization (STIPO) domains for all groups. Mean values for the STIPO domains (range 1–5) with standard deviations shown as error bars with 95% confidential intervals are displayed. BPD, borderline personality disorder; FEP, first-episode psychosis; HC, healthy controls; UHR, ultra-high risk for psychosis.

The scores on the seven domains of the STIPO are shown in Fig. 1. The distributions of the seven domains were not identical between groups (χ²(3) = 31.609–63.522, *P* < 0.001). Although the overall STIPO domain ‘Identity’ did not show any significant differences between the diagnostic groups, the subdomain ‘Sense of self – coherence and continuity’ was significantly impaired in the FEP compared with the UHR group (*U* = 224.00, *P* = 0.021) and in the FEP compared with the BPD group (*U* = 274.00, *P* = 0.04). Object relations (domain 2), especially the internal working model of relationships, were found to be significantly better in the UHR group compared with the BPD (*U* = 206.50, *P* = 0.017), but no significant differences were found between other groups. Primitive defences were significantly more pronounced in participants with FEP than those at UHR (*U* = 205.5, *P* = 0.009). Participants with BPD had higher overall aggression scores compared with those at UHR (*U* = 206.00, *P* = 0.026) and higher scores in aggression directed at the self (*U* = 204.00, *P* = 0.003) and at others (*U* = 227.50, *P* = 0.009) compared with FEP individuals. Reality testing showed significant differences between all diagnostic groups: FEP and BPD (*U* = 83.50, *P* < 0.001); FEP and UHR (*U* = 140.50, *P* < 0.001); and UHR and BPD (*U* = 194.50, *P* = 0.015).

#### Level of Personality Functioning Scale (LPFS)

**Fig. 2:**
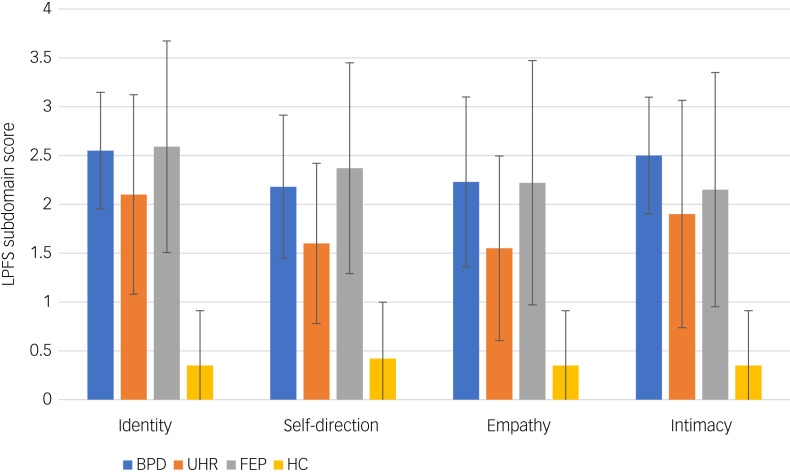
Bar chart of Level of Personality Functioning Scale (LPFS) domains for all groups. Mean values for the LPFS domains (range 0–4) with standard deviations shown as error bars with 95% confidence intervals are displayed. BPD, borderline personality disorder; FEP, first-episode psychosis; HC, healthy controls; UHR, ultra-high risk for psychosis.

Mean levels of personality functioning are shown in Fig. 2. The distribution of the four subdomains of the LPFS were not identical across all groups (χ²(3) = 37.726–50.754, *P* < 0.001). All patient groups showed significantly more impairment, with higher scores in all four subdomains compared with healthy controls (*P* = 0.001). Self-direction was significantly better in the UHR compared with the FEP group (*U* = 156.00, *P* = 0.011) and the BDP group (*U* = 138.00, *P* = 0.026). Empathy was significantly higher in individuals at UHR than in those with BPD (*U* = 140.50, *P* = 0.033), but no significant differences between groups were found for identity and intimacy.

### Level of self-disorders

The FEP group showed the highest level of self-disturbance as measured by the EASE (mean 21.21, s.d. = 8.187), whereas the BPD group showed the lowest level (mean 14.71, s.d. = 7.357), with the UHR group in between (mean 17.08, s.d. = 6.953). Group differences in self-disturbances were significant between FEP and BPD (*P* < 0.019), as well as between all diagnostic groups and healthy controls (*P* > 0.001).

### The relationship between levels of personality functioning and basic self-disorders

Correlation analysis showed significant large positive correlations of the LPFS domains with the STIPO domains ‘Identity’ and ‘Object relations’ (*ρ* = 0.738 to *ρ* = 0.858, *P* < 0.001, *n* = 95).

Moderate to large positive correlations of EASE total score with all STIPO domains and STIPO total score were found (*ρ* = 0.392–0.763, *P* < 0.001; highest correlations with STIPO domain ‘Reality testing’ and total score).

### Clustering of patients according to personality functioning

**Fig. 3:**
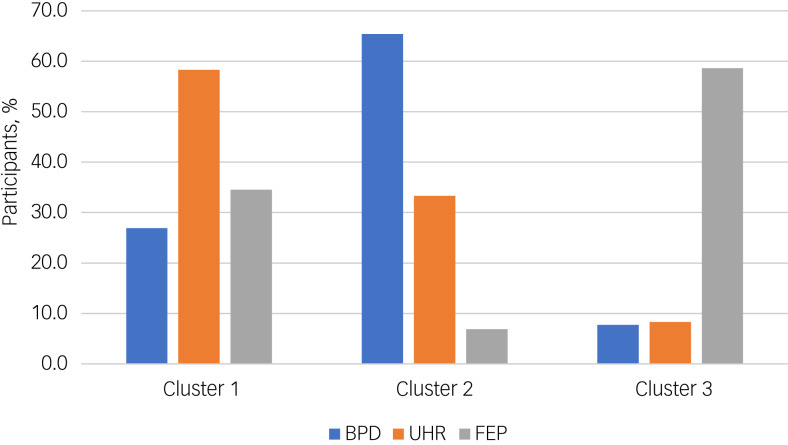
Bar chart of the distribution (%) of diagnostic groups in the three clusters derived from the cluster analysis based on the seven Structured Interview of Personality Organization domains. BPD, borderline personality disorder; FEP, first-episode psychosis; UHR, ultra-high risk for psychosis.

The hierarchical cluster analysis of the three patient groups (FEP, UHR, BPD) based on the seven STIPO domains revealed that the greatest increase in heterogeneity is reached with a two-cluster solution, and the next highest with a three-cluster solution (Supplementary Fig. 4, available at https://dx.doi.org/10.1192/bjo.2023.530). These three clusters differed significantly with respect to overall level of personality functioning (χ²(2) = 42.596, *P* < 0.001). Cluster 1 includes patients with only moderate impairment in overall personality functioning (mean 3.94; s.d. = 0.63). Cluster 2 includes patients with slightly better functioning (mean 4.78, s.d. = 0.42) and cluster 3 shows those with the most severe impairment in personality functioning (mean 5.48, s.d. = 0.68) (for more detailed results for the STIPO domains see Supplementary Table 4). Regarding the distribution of the diagnostic groups, more than half of the UHR participants could be found in cluster 1, while about two-thirds of participants with BPD were grouped in cluster 2, and more than half of FEP participants in cluster 3 (Fig. 3). Significant differences between clusters were also found in relation to mean EASE scores (*F*(2,74) = 5.187, *P* = 0.008). High levels of basic self-disorders were found in cluster 1 (mean 19.33, s.d. = 9.17) and cluster 3 (mean 20.57, s.d. = 5.77), whereas cluster 2 showed significantly lower levels (mean 14.08, s.d. = 6.68).

### Ultra-high risk (UHR) and borderline personality disorder (BPD) interaction

Eight individuals with BPD had elevated PQ-16 scores, which might point to an at-risk mental state for psychosis, but no significant difference in STIPO overall scores and domains were found compared with participants with BPD without elevated PQ-16 scores. No differences in overall STIPO-rated personality functioning were found between UHR individuals with comorbid BPD (*n* = 6) and without comorbid BPD (*n* = 18), but UHR individuals without comorbid BPD showed a better identity integration (*P* = 0.015), with a better sense of others (*P* = 0.018) and lower scores for primitive defences (*P* = 0.015) compared with UHR individuals with comorbid BPD.

## Discussion

### Level of personality functioning

Our results demonstrate that psychosis spectrum disorders are associated with impaired personality functioning. Both UHR and FEP individuals were found to have moderate to severe deficits in personality functioning as measured by the STIPO and the LPFS when compared with healthy controls. Furthermore, individuals with manifest psychosis (FEP) showed significantly worse overall personality functioning than individuals at risk for psychosis (UHR), confirming previous data on personality functioning in different stages of psychotic disorders.^26^

Our results show no significant differences between individuals at risk for or with psychosis (UHR/FEP) and individuals with BPD in the overall level of personality functioning. The hypothesis derived from psychodynamic theory that individuals on the psychosis spectrum (UHR and FEP) have a psychotic personality organisation which is characterised by more impaired personality functioning in all domains (severe identity diffusion, extensive use of primitive defence mechanisms) and impaired reality testing (corresponding to a loss of differentiation between self and object representations) compared with individuals on the borderline disorder spectrum, who have a borderline personality organisation with less severely impaired personality functioning, could therefore not be confirmed in our sample.^18,49^ These results might reflect dimensional variations of severity within psychotic disorders and BPD.^50^ In ICD-11, the distinction between schizophrenia and ultra-high risk of psychosis (e.g. schizotypal disorder) is based on the intensity of symptoms, and severe personality disorders (e.g. low borderline level) can also have psychotic features (transient dissociative or psychosis-like symptoms).^50^ The BPD sample in this study was mainly recruited from in-patient settings, suggesting a severely ill sample,^51^ which is reflected in comparatively low levels of personality functioning (mean 4.70, s.d. = 0.542) and relatively high levels of self-disorders (EASE: mean 14.71, s.d. = 7.357).

### Domains of personality functioning

The pattern of personality functioning on the LPFS and the seven domains of the STIPO points to several noteworthy differences between the diagnostic groups.

The UHR group showed better self-functioning (self-direction) than the BPD and FEP groups. A higher quality of object relations with better internal working models of relationships and more empathy was also found in the UHR compared with the BPD group. Additionally, UHR individuals without comorbid BPD showed a better identity integration with a better sense of others and less use of primitive defences compared with UHR individuals with comorbid BPD. Furthermore, our results show a significantly higher level of aggression directed against self and others in participants with BPD compared with those at UHR and with FEP. These results contradict the stereotype that psychosis is associated with a greater degree of violence.^52^ Psychoanalytic object relations theory characterises borderline personality organisation as having deficiencies in developing an integrated view of self and significant others as well as developmental difficulties in integrating and modulating aggressive impulses, leading to the observable deficits in object relations.^17^ The problems in interpersonal relationships appear to be pathognomonic for BPD and according to our results apparently not specific for UHR individuals. Interestingly, a recent study argues that psychotic symptoms are also common in people with clinically diagnosed BPD^53^ and other studies found that the comorbidity with BPD diagnoses or BPD features did not influence the risk of short-term transition to psychosis in UHR patients.^54^ The domain of primitive defences was more pronounced in participants with FEP than in those at UHR. According to psychodynamic theoretical and empirical findings, the more extensive use of primitive defences such as projective identification, splitting or primitive denial characterises the defence structure in manifest psychosis.^55^ Not surprisingly, significant differences were found in reality testing and perceptual distortions between the FEP and all other groups as well as between the UHR and BPD groups, with the loss of reality testing being a cardinal aspect of psychotic functioning.^18^

### Self-disorders

Moderate to high levels of self-disorders as measured by the EASE were found in all diagnostic groups. Participants with manifest psychosis (FEP) showed significantly higher levels of disturbance of the basic self than those with non-psychotic disorders (BPD). The extent of self-disorders in UHR patients was intermediate between that of patients with BPD and with FEP. Like our study, recent reviews^22,24^ also found more self-disorders in UHR groups compared with healthy controls. Our results empirically support the hypothesis that the level of self affected in psychosis is more ‘basic’ as measured by the EASE than the higher-level identity disturbances pathognomonic for BPD as measured by the overall STIPO domain ‘Identity’, which would correspond to disturbances of the ‘narrative self’.^25^ Notably, the STIPO subdomain ‘Sense of self – coherence and continuity’, which covers more ‘basic’ aspects of the self such as the consistency of the sense of self in different social situations and over time, did show significant differences between the FEP and BPD groups. Nevertheless, with a mean score of 14.71 on the EASE, the BPD group showed remarkably high levels of basic self-disturbance, given recent data that point to a very high specificity for the presence of a schizophrenia spectrum disorder for EASE scores >11.^56^ In this sense, it has been argued that people with BPD with high levels of self-disturbance might be better classified as belonging on the schizophrenia spectrum.^10,53,57^ However, it could alternatively be argued that the EASE might not effectively discriminate between severe identity disturbances seen in low-level BPD patients and in schizophrenia spectrum disorders. The assessment of the nature of self- or identity-disturbances thus is of great diagnostic and clinical importance and the sole reliance on self-reports seems to be insufficient for these purposes,^58^ underlining the need for careful and thorough psychopathological investigations.^25^

### Cluster analysis

The hierarchical cluster analysis based on the seven STIPO domains yielded three clusters that differed significantly in terms of both overall level of personality functioning and self-disturbances. Cluster analysis identified a group of individuals with FEP have a significantly higher level of personality functioning, pointing to the above-mentioned dimensional variations within a diagnostic category. It seems that psychotic disorders can occur with all levels of personality functioning, from a high level to very severe impairment. Furthermore, cluster analysis showed some UHR participants to be more closely related to participants with BPD who had rather severe impairment in personality functioning but only little basic self-disturbance and intact reality testing, whereas others aggregated together with the higher-functioning FEP participants who had more anomalous self-experiences and deficits in reality testing.

The period at ultra-high risk and the first 5 years after the onset of psychosis are a critical periods during which early interventions can potentially significantly influencing the course of illness.^59^ Awareness of the importance and effectiveness of psychotherapeutic and other interventions that incorporate related concepts (e.g. metacognitive insight and reflection therapy^60^) for people with psychosis spectrum disorders is growing.^60–62^ The differential diagnosis between a schizophrenia spectrum disorder and BPD is often challenging and existing structured interviews might not adequately capture the core psychopathological phenomena that allow an accurate diagnosis to be made.^53,57^ To improve the differential diagnosis, a comprehensive diagnostic evaluation in early detection should include the assessment of personality functioning and self-disorders. This might also help to identify focuses for psychotherapeutic treatment and provide a clinically meaningful measure of therapeutic change.^60–62^

### Limitations

Limitations of the current study include its cross-sectional design and the relatively small sample, which was due to the time-consuming assessment procedures. The FEP group in our study included both affective and non-affective FEP, even though there is evidence that self-disorders are more common in schizophrenia than in affective psychosis.^22^ Personality functioning assessment (STIPO/LPFS) covered the previous 5 years, which may be too long to capture recent loss of personality functioning due to the onset of psychotic experiences in the UHR and FEP samples. Self-disorders (as measured with the EASE) do not reflect ‘psychosis’ but schizophrenia (susceptibility). Nevertheless, in this study the EASE was not able to distinguish sufficiently between low-grade borderline personality organisation and schizophrenia susceptibility.

Further longitudinal studies are needed to investigate the complex relationship between personality pathology and psychotic disorders, including the predictive value of impairments in personality functioning and self-disorders for identifying UHR individuals who eventually develop a psychotic disorder and for the development of personality functioning over time in psychotic disorders.

## Supporting information

Gruber et al. supplementary material Gruber et al. supplementary material

## Data Availability

The data that support the findings of this study are available from the corresponding author, V.B., on reasonable request.

## References

[1] Newton-Howes G, Tyrer P, North B, Yang M. The prevalence of personality disorder in schizophrenia and psychotic disorders: systematic review of rates and explanatory modelling. Psychol Med 2008; 38: 1075–82. 18070369 10.1017/S0033291707002036

[2] Simonsen E, Newton-Howes G. Personality pathology and schizophrenia. Schizophr Bull 2018; 44: 1180–4. 29688529 10.1093/schbul/sby053 PMC6192496

[3] Begemann MJH, Boyette L-L, Kwast AK, Sommer IEC. Personality across the psychosis continuum: a fine-grained perspective. Schizophr Bull Open 2020; 1(1): sgaa064.

[4] Franquillo AC, Guccione C, Angelini G, Carpentieri R, Ducci G, Caretti V. The role of personality in schizophrenia and psychosis: a systematic review. Clin Neuropsychiatry 2021; 18: 28–40. 34909018 10.36131/cnfioritieditore20210103 PMC8629049

[5] Parnas J, Zandersen M. Self and schizophrenia: current status and diagnostic implications. World Psychiatry 2018; 17: 220–1. 29856572 10.1002/wps.20528 PMC5980564

[6] Sass LA, Parnas J. Schizophrenia, consciousness and the Self. Schizophr Bull 2003; 29: 427–44. 14609238 10.1093/oxfordjournals.schbul.a007017

[7] Jobe TH, Harrow M. Schizophrenia course, long-term outcome, recovery, and prognosis. Curr Dir Psychol Sci 2010; 19: 220–5.

[8] Carrión RE, McLaughlin D, Goldberg TE, Auther AM, Olsen RH, Olvet DM, et al. Prediction of functional outcome in individuals at clinical high risk for psychosis. JAMA Psychiatry 2013; 70: 1133–42. 24006090 10.1001/jamapsychiatry.2013.1909 PMC4469070

[9] Balaratnasingam S, Janca A. Normal personality, personality disorder and psychosis. Curr Opin Psychiatry 2015; 28: 30–4. 25415496 10.1097/YCO.0000000000000124

[10] Zandersen M, Henriksen MG, Parnas J. A recurrent question: what is borderline? J Pers Disord 2019; 33: 341–69. 29469662 10.1521/pedi_2018_32_348

[11] Cavelti M, Thompson K, Chanen AM, Kaess M. Psychotic symptoms in borderline personality disorder: developmental aspects. Curr Opin Psychol 2021; 37: 26–31. 32771980 10.1016/j.copsyc.2020.07.003

[12] Montag C. Zur Differenzialdiagnostik von Borderline-Persönlichkeitsstörung und Schizophrenien anhand von psychotischer Positivsymptomatik [On the differential diagnosis of borderline personality disorder and schizophrenia based on positive psychotic symptoms]. PTT 2021; 25: 83–95.

[13] American Psychiatric Association. Diagnostic and Statistical Manual of Mental Disorder (5th edn) (DSM-5). APA Publishing, 2013.

[14] World Health Organization. ICD-11 for Mortality and Morbidity Statistics. WHO, 2019 (https://icd.who.int/en).

[15] Gruber M, Doering S, Blüml V. Personality functioning in anxiety disorders. Curr Opin Psychiatry 2020; 33: 62–9. 31790374 10.1097/YCO.0000000000000556

[16] Kernberg O. Severe Personality Disorders: Psychotherapeutic Strategies. Yale University Press, 1984.

[17] Kernberg OF. Borderline Conditions and Pathological Narcissism. Jason Aronson, 1975.

[18] Kernberg OF. Psychotic personality structure. Psychodyn Psychiatry 2019; 47: 353–72. 31913791 10.1521/pdps.2019.47.4.353

[19] Nelson B. Varieties of self-disturbance: a prism through which to view mental disorder. Early Interv Psychiatry 2013; 7: 231–4. 23879831 10.1111/eip.12080

[20] Nordgaard J, Henriksen MG, Jansson L, Handest P, Møller P, Rasmussen AR, et al. Disordered selfhood in schizophrenia and the examination of anomalous self-experience: accumulated evidence and experience. Psychopathology 2021; 54: 275–81. 34384082 10.1159/000517672 PMC8686724

[21] Nelson B, Thompson A, Chanen AM, Amminger GP, Yung AR. Is basic self-disturbance in ultra-high risk for psychosis (‘prodromal’) patients associated with borderline personality pathology? Early Interv Psychiatry 2013; 7: 306–10. 23347769 10.1111/eip.12011

[22] Raballo A, Poletti M, Preti A, Parnas J. The self in the spectrum: a meta-analysis of the evidence linking basic self-disorders and schizophrenia. Schizophr Bull 2021; 47: 1007–17. 33479736 10.1093/schbul/sbaa201 PMC8266610

[23] Koren D, Tzivoni Y, Schalit L, Adres M, Reznik N, Apter A, et al. Basic self-disorders in adolescence predict schizophrenia spectrum disorders in young adulthood: a 7-year follow-up study among non-psychotic help-seeking adolescents. Schizophr Res 2020; 216: 97–103. 31889574 10.1016/j.schres.2019.12.022

[24] Henriksen MG, Raballo A, Nordgaard J. Self-disorders and psychopathology: a systematic review. Lancet Psychiatry 2021; 8: 1001–12. 34688345 10.1016/S2215-0366(21)00097-3

[25] Zandersen M, Parnas J. Identity disturbance, feelings of emptiness, and the boundaries of the schizophrenia spectrum. Schizophr Bull 2019; 45: 106–13. 29373752 10.1093/schbul/sbx183 PMC6293220

[26] Uzdawinis D, Edel MA, Özgürdal S, Von Haebler D, Hauser M, Witthaus H, et al. Operationalisierte psychodynamische diagnostik (OPD) bei patienten im schizophrenen prodromalstadium – Eine explorative studie [Operationalized psychodynamic diagnostics (OPD) in patients in a prodromal state of schizophrenia – an explorative study]. Z Psychosom Med Psychother 2010; 56: 150–62. 20623460 10.13109/zptm.2010.56.2.150

[27] Yung AR, Yung AR, Pan Yuen H, Mcgorry PD, Phillips LJ, Kelly D, et al. Mapping the onset of psychosis: the comprehensive assessment of at-risk mental states. Aust New Zeal J Psychiatry 2005; 39: 964–71. 10.1080/j.1440-1614.2005.01714.x 16343296

[28] Schultze-Lutter F, Addington J, Ruhrmann S, Klosterkötter J. Schizophrenia Proneness Instrument, Adult Version (SPI-A). Giovanni Fioriti Editore, 2007 (https://www.fioritieditore.com/wp-content/uploads/2016/06/Schultze-Lutter.doc).

[29] Schultze-Lutter F, Michel C, Schmidt SJ, Schimmelmann BG, Maric NP, Salokangas RKR, et al. EPA guidance on the early detection of clinical high risk states of psychoses. Eur Psychiatry 2015; 30: 405–16. 25735810 10.1016/j.eurpsy.2015.01.010

[30] Breitborde NJK, Srihari VH, Woods SW. Review of the operational definition for first-episode psychosis. Early Interv Psychiatry 2009; 3: 259–65. 22642728 10.1111/j.1751-7893.2009.00148.x PMC4451818

[31] Wittchen H-U, Fydrich T, Zaudig M, Fydrich T. SKID: Strukturiertes Klinisches Interview für DSM-IV; Achse I und II. Achse II: Persönlichkeitsstörungen. SKID-II [SCID: The Structured Clinical Interview for DSM-IV; Axes I and II. Axis II: Personality Disorders]. Hogrefe, 1997.

[32] Kim JS, Baek JH, Choi JS, Lee D, Kwon JS, Hong KS. Diagnostic stability of first-episode psychosis and predictors of diagnostic shift from non-affective psychosis to bipolar disorder: A retrospective evaluation after recurrence. Psychiatry Res 2011; 188: 29–33. 21056477 10.1016/j.psychres.2010.09.017

[33] Quattrone D, Di Forti M, Gayer-Anderson C, Ferraro L, Jongsma HE, Tripoli G, et al. Transdiagnostic dimensions of psychopathology at first episode psychosis: findings from the multinational EU-GEI study. Psychol Med 2019; 49: 1378–91. 30282569 10.1017/S0033291718002131 PMC6518388

[34] Peralta V, Moreno-Izco L, García de Jalón E, Sánchez-Torres AM, Janda L, Peralta D, et al. Prospective long-term cohort study of subjects with first-episode psychosis examining eight major outcome domains and their predictors: study protocol. Front Psychiatry 2021; 12: 643112. 10.3389/fpsyt.2021.643112 PMC8017172 33815175

[35] Kay SR, Fiszbein A, Opler LA. The Positive and Negative Syndrome Scale (PANSS) for schizophrenia. Schizophr Bull 1987; 13: 261–76. 3616518 10.1093/schbul/13.2.261

[36] Ising HK, Veling W, Loewy RL, Rietveld MW, Rietdijk J, Dragt S, et al. The validity of the 16-item version of the Prodromal Questionnaire (PQ-16) to screen for ultra high risk of developing psychosis in the general help-seeking population. Schizophr Bull 2012; 38: 1288–96. 22516147 10.1093/schbul/sbs068 PMC3713086

[37] Franke G. BSI. Brief Symptom Inventory – Deutsche Version. Manual. Beltz, 2000.

[38] Clarkin JF, Caligor E, Stern B, Kernberg OF. Structured Interview of Personality Organization (STIPO). Weill Medical College of Cornell University, 2007 (https://istfp.org/wp-content/uploads/2019/08/Structured-Interview-of-Personality-Organization.pdf).

[39] Doering S, Burgmer M, Heuft G, Menke D, Bäumer B, Lübking M, et al. Reliability and validity of the German version of the Structured Interview of Personality Organization (STIPO). BMC Psychiatry 2013; 13: 210. 23941404 10.1186/1471-244X-13-210 PMC3751705

[40] Stern BL, Caligor E, Clarkin JF, Critchfield KL, Horz S, MacCornack V, et al. Structured Interview of Personality Organization (STIPO): preliminary psychometrics in a clinical sample. J Pers Assess 2010; 92: 35–44. 20013454 10.1080/00223890903379308

[41] Bender DS, Morey LC, Skodol AE. Toward a model for assessing level of personality functioning in DSM–5, Part I: a review of theory and methods. J Pers Assess 2011; 93: 332–46. 22804672 10.1080/00223891.2011.583808

[42] Zimmermann J, Benecke C, Bender DS, Skodol AE, Schauenburg H, Cierpka M, et al. Assessing DSM-5 level of personality functioning from videotaped clinical interviews: a pilot study with untrained and clinically inexperienced students. J Pers Assess 2014; 96: 397–409. 24224740 10.1080/00223891.2013.852563

[43] Di Pierro R, Gargiulo I, Poggi A, Madeddu F, Preti E. The level of personality functioning scale applied to clinical material from the Structured Interview of Personality Organization (STIPO): utility in detecting personality pathology. J Pers Disord 2020; 34(suppl C): 1–15. 10.1521/pedi_2020_34_472 32163025

[44] Parnas J, Møller P, Kircher T, Thalbitzer J, Jansson L, Handest P, et al. EASE: Examination of Anomalous Self-Experience. Psychopathology 2005; 38: 236–58. 16179811 10.1159/000088441

[45] Møller P, Haug E, Raballo A, Parnas J, Melle I. Examination of anomalous self-experience in first-episode psychosis: Interrater reliability. Psychopathology 2011; 44: 386–90. 21847006 10.1159/000325173

[46] Loewy RL, Bearden CE, Johnson JK, Raine A, Cannon TD. The Prodromal Questionnaire (PQ): preliminary validation of a self-report screening measure for prodromal and psychotic syndromes. Schizophr Res 2005; 79: 117–25. 16276559

[47] Blashfield RK. Mixture model tests of cluster analysis: accuracy of four agglomerative hierarchical methods. Psychol Bull 1976; 83: 377–88.

[48] American Psychiatric Association. Diagnostic and Statistical Manual of Mental Disorders (4th edn, text revision) (DSM-IV-TR). APA, 2000.

[49] Dauphin J. Differentiation between schizophreniform configurations and psychotic personality structures. Psychodyn Psychiatry 2017; 45: 187–216. 28590204 10.1521/pdps.2017.45.2.187

[50] Bach B, First MB. Application of the ICD-11 classification of personality disorders. BMC Psychiatry 2018; 18: 351. 30373564 10.1186/s12888-018-1908-3 PMC6206910

[51] Kjær JN, Biskin R, Vestergaard C, Munk-Jørgensen P. All-Cause mortality of hospital-treated borderline personality disorder: a nationwide cohort study. J Pers Disord 2020; 34: 723–35. 30307824 10.1521/pedi_2018_32_403

[52] Yang LH, Anglin DM, Wonpat-Borja AJ, Opler MG, Greenspoon M, Corcoran CM. Public stigma associated with psychosis risk syndrome in a college population: implications for peer intervention. Psychiatr Serv 2013; 64: 284–8. 23450386 10.1176/appi.ps.003782011 PMC3672401

[53] Zandersen M, Parnas J. Exploring schizophrenia spectrum psychopathology in borderline personality disorder. Eur Arch Psychiatry Clin Neurosci 2020; 270: 969–78. 31289925 10.1007/s00406-019-01039-4 PMC7599140

[54] Thompson A, Nelson B, Bechdolf A, Chanen AM, Domingues I, Mcdougall E, et al. Borderline personality features and development of psychosis in an “ultra High Risk” (Uhr) population: a case control study. Early Interv Psychiatry 2012; 6: 247–55. 22672502 10.1111/j.1751-7893.2012.00365.x

[55] Leichsenring F. Primitive defense mechanisms in schizophrenics and borderline patients. J Nerv Ment Dis 1999; 187: 229–36. 10221556 10.1097/00005053-199904000-00006

[56] Nordgaard J, Berge J, Rasmussen AR, Sandsten KE, Zandersen M, Parnas J. Are self-disorders in schizophrenia expressive of a unifying disturbance of subjectivity: a factor analytic approach. Schizophr Bull 2023; 49: 144–50. 36073251 10.1093/schbul/sbac123 PMC9809994

[57] Zandersen M, Parnas J. Borderline personality disorder or a disorder within the schizophrenia spectrum? A psychopathological study. World Psychiatry 2019; 18: 109–10. 30600641 10.1002/wps.20598 PMC6313234

[58] Meisner MW, Lenzenweger MF, Bach B, Vestergaard M, Petersen LS, Haahr UH, et al. Exploring identity disturbance and psychotic spectrum symptoms as predictors of borderline and schizotypal personality disorders. Psychopathology 2021; 54: 193–202. 34058737 10.1159/000516209

[59] Singh SP, Mohan M, Giacco D. Psychosocial interventions for people with a first episode psychosis: between tradition and innovation. Curr Opin Psychiatry 2021; 34: 460–6. 34282104 10.1097/YCO.0000000000000726

[60] Schweitzer RD, Greben M, Bargenquast R. Long-term outcomes of metacognitive narrative psychotherapy for people diagnosed with schizophrenia. Psychol Psychother Theory Res Pract 2017; 90: 668–85. 10.1111/papt.12132 28544223

[61] Lempa G, von Haebler D, Montag C. Psychodynamische Psychotherapie der Schizophrenien: Ein Manual [Psychodynamic Psychotherapy for Schizophrenia: A Manual]. Psychosozial-Verlag, 2017.

[62] Müller H, Laier S, Bechdolf A. Evidence-based psychotherapy for the prevention and treatment of first-episode psychosis. Eur Arch Psychiatry Clin Neurosci 2014; 264: 17–25. 10.1007/s00406-014-0538-0 25261211

