# Supplementary material for: Personality functioning and self-disorders in individuals at ultra-high risk for psychosis, with first-episode psychosis and with borderline personality disorder
Source: BJPsych Open. 2023 Aug 11;9(5):e150. doi: 10.1192/bjo.2023.530 (PMC10594090; doi:10.1192/bjo.2023.530)
Supplement: Gruber et al. supplementary material [file S2056472423005306sup001.docx]

**Supplementary Data - Personality functioning and self-disorders in individuals at ultra-high risk for psychosis, with first-episode psychosis and with borderline personality disorder**

**Figure 4** Hierarchical clustering result dendrogram. Hierarchical clustering (Ward's method), based on the seven STIPO domains, results shown as a dendrogram of 79 patients at UHR, with FEP or BPD. The x-axis shows the study-specific distances between the clusters.


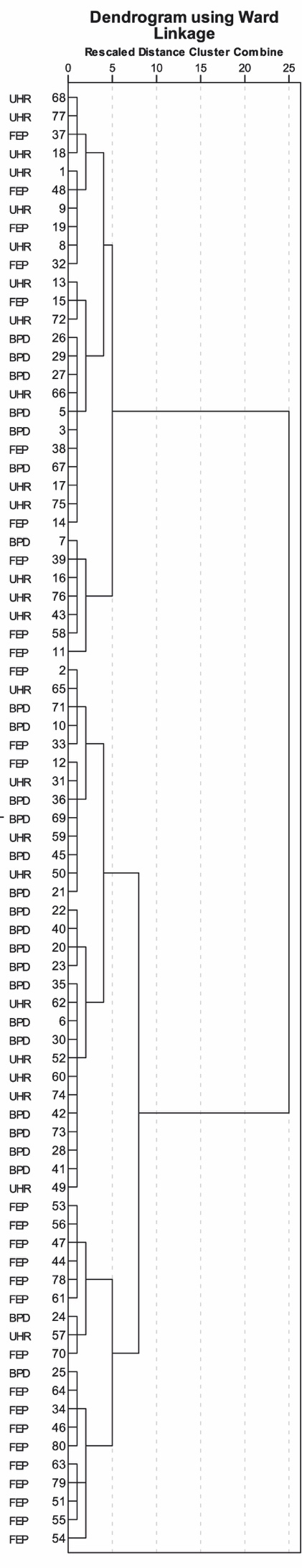


**Table 4** Results of the cluster analysis, Mean values of STIPO domains (range between 1 and 5) with standard deviations (SD) with 95% confidential intervals are shown. Test statistics of the cluster differences as calculated with the Mann-Whitney-U-Test are indicated.

| Ward Method | | Identity | Object relation | Primitive Defenses | Coping / rigiditiy | Aggression | Moral issues | Reality testing |
| --- | --- | --- | --- | --- | --- | --- | --- | --- |
| Cluster 1 | Mean | 2.71 | 2.42 | 2.87 | 2.81 | 2.45 | 1.68 | 3.23 |
|  | N | 31.00 | 31.00 | 31.00 | 31.00 | 31.00 | 31.00 | 31.00 |
|  | SD | 0.46 | 0.76 | 0.50 | 0.70 | 1.03 | 0.65 | 1.06 |
| Cluster 2 | Mean | 3.79 | 3.48 | 3.90 | 3.93 | 3.28 | 2.45 | 2.83 |
|  | N | 29.00 | 29.00 | 29.00 | 29.00 | 29.00 | 29.00 | 29.00 |
|  | SD | 0.49 | 0.57 | 0.49 | 0.75 | 0.45 | 0.78 | 0.80 |
| Cluster 3 | Mean | 4.21 | 3.84 | 4.74 | 4.53 | 3.32 | 2.63 | 4.53 |
|  | N | 19.00 | 19.00 | 19.00 | 19.00 | 19.00 | 19.00 | 19.00 |
|  | SD | 0.79 | 0.83 | 0.45 | 0.70 | 1.00 | 1.12 | 0.61 |
| Test statistics | |  |  |  |  |  |  |  |
|  |  |  |  |  |  |  |  |  |
| 1 vs. 2 | U | 77.00 | 140.00 | 89.50 | 131.50 | 213.00 | 217.50 | 362.00 |
|  | p | 0.00 | 0.00 | 0.00 | 0.00 | 0.00 | 0.00 | .17 |
| 1 vs. 3 | U | 44.00 | 70.00 | 5.00 | 34.50 | 158.50 | 145.50 | 98.50 |
|  | p | 0.00 | 0.00 | 0.00 | 0.00 | 0.00 | 0.00 | 0.00 |
| 2 vs. 3 | U | 186.50 | 203.50 | 79.00 | 156.00 | 259.00 | 253.50 | 33,50 |
|  | p | 0.03 | 0.09 | 0.00 | 0.01 | 0.69 | 0.62 | 0.00 |
